# Experiences of Diagnosis, Symptoms, and Use of Reliever Inhalers in Patients With Asthma and Concurrent Inducible Laryngeal Obstruction or Breathing Pattern Disorder: Qualitative Analysis of a UK Asthma Online Community

**DOI:** 10.2196/44453

**Published:** 2023-08-14

**Authors:** Catrin Byrne, Paul E Pfeffer, Anna De Simoni

**Affiliations:** 1 Wolfson Institute of Population Health Queen Mary University of London London United Kingdom; 2 Barts Health NHS Trust London United Kingdom; 3 Wolfson Institute of Population Health Asthma UK Centre for Applied Research Queen Mary University of London London United Kingdom

**Keywords:** asthma, breathing pattern disorder, inducible laryngeal obstruction, BPD, ILO, short-acting beta-agonist, salbutamol, breathing disorder, breathing, chest tightness, community, symptoms, diagnosis

## Abstract

**Background:**

Breathing pattern disorders (BPDs) and inducible laryngeal obstruction (ILO) cause similar symptoms to asthma, including dyspnea and chest tightness, with an estimated prevalence of up to one-fifth of patients with asthma. Both conditions can be comorbid with asthma, and there is evidence that they are misdiagnosed and mistreated as asthma.

**Objective:**

This study aims to explore whether the symptoms of ILO and BPD were topics of discussion in a UK asthma online health community and patient experiences of diagnosis and treatment, in particular their use of reliever inhalers.

**Methods:**

A qualitative thematic analysis was performed with posts from an asthma community between 2018 and 2022. A list of key ILO or BPD symptoms was created from the literature. Posts were identified using the search terms “blue inhaler” and “breath” and included if describing key symptoms. Discussion threads of included posts were also analyzed.

**Results:**

The search retrieved a total of 1127 relevant posts: 1069 written by 302 users and 58 posted anonymously. All participants were adults, except 2 who were parents writing about their children. Sex and age were only available for 1.66% (5/302; 3 females and 2 males) and 9.93% (30/302) of participants (27 to 73 years old), respectively. The average number of posts written by each participant was 3.54 (range 1-63). Seven participants wrote >20 posts each. Participants experiencing undiagnosed ILO or BPD symptoms, whether or not comorbid with asthma, expressed frustration with the “one-size-fits-all” approach to diagnosis, as many felt that their asthma diagnosis did not fully explain symptoms. Some suspected or were formally diagnosed with BPD or ILO, the latter reporting relief on receiving a diagnosis and appropriate management. Participants showed awareness of their inappropriate salbutamol use or overuse due to lack of effect on symptoms. BPD and ILO symptoms were frequently comorbid with asthma. The asthma online community was a valuable resource: engagement with peers not only brought comfort but also prompted action with some going back to their clinicians and reaching a diagnosis and appropriate management.

**Conclusions:**

Undiagnosed ILO and BPD symptoms and lack of effects of asthma treatment were topics of discussion in an asthma online community, caused distress and frustration in participants, and affected their relationship with health care professionals, showing that patients experiencing BPD and ILO have unmet needs. Clinicians’ education on BPD and ILO diagnosis and management, as well as increased access to appropriate management options, such as respiratory physiotherapy and speech and language therapy, are warranted particularly in primary care. Qualitative evidence that engagement with the online community resulted in patients taking action going back to their clinicians and reaching a diagnosis of ILO and BPD prompts future research on online peer support from an established online health community as a self-management resource for patients.

## Introduction

Asthma is a common disease, with approximately 12% of the UK population having received a diagnosis of asthma at some point in their lifetime [1]. The most common symptoms of asthma are cough and shortness of breath; however, there are other conditions causing similar symptoms, such as breathing pattern disorders (BPD) and inducible laryngeal obstruction (ILO).

BPD, also known as dysfunctional breathing, is concurrently present in around one-thirds of women with asthma and one-fifth of men with asthma in the United Kingdom [2], and also many people without asthma. BPD can be responsible for symptoms attributed to asthma, including dyspnea and chest tightness [3]. BPD is commonly misdiagnosed as asthma; 1 study found that 10% of people diagnosed with asthma actually had BPD and no asthma [4]. Patients with BPD often have more chronic breathlessness rather than the classical episodic breathlessness of asthma. The most reliable method of diagnosing BPD is a comprehensive respiratory physiotherapy assessment [5]. As this is not routinely available, quick patient questionnaires and quick visual assessments have been developed [5,6]. However, there is no evidence to suggest these tools are used routinely in general practice to assess for the presence of concurrent BPD in patients with asthma. Making the correct diagnosis is important as BPD responds to breathing exercises and respiratory physiotherapy rather than asthma pharmacotherapy [7-9].

ILO, also called vocal cord dysfunction, causes cough, throat tightness, and dyspnea [10]. The condition compromises reversible, inappropriate narrowing of the larynx, in response to external triggers such as smells or odors, and in some patients exercise [10,11]. Although more research is needed into the incidence of ILO, individual studies have reported findings such as 19% of patients with asthma also having undiagnosed ILO [12]. Inversely, a study of 95 patients diagnosed with ILO found that 56% had concurrent asthma [13]. The gold standard for diagnosis of ILO is direct visualization of the vocal cords during an acute episode [13,14]. Laryngoscopy is a relatively resource-intensive procedure and is not appropriate for diagnosis in primary care—as an alternative, a simple symptom-scoring measure called the Pittsburgh VCD index has been developed to identify patients with likely ILO [15]. However, this tool is not routinely used in primary care where the majority of patients with asthma are managed. One study of clinicians in the United Kingdom involved in respiratory and airway medicine found that 83% had not heard of or knew little about ILO [16]. The recommended management of ILO involves speech and language therapy, physiotherapy, management of potential triggers, and smoking cessation [17]. However, ILO is often misdiagnosed as asthma, and this misdiagnosis can result in inappropriate treatment with asthma medication, including inhalers and oral steroids [13,14].

Treatment of BPD and ILO with asthma medication not only results in poor symptom control [13] but if used inappropriately, short-acting beta-agonists can have negative consequences, such as psychological dependence on inhalers [18,19], side effects such as arrhythmias, headaches, hypokalemia, and muscle spasms and can worsen airway hyperresponsiveness [20]. From our experience, patients using but not responding to inhaled therapy, due to incorrect diagnosis, also experience psychological distress from nonresponse to treatment. Inappropriate prescription of inhalers also leads to increased public cost, with over £9,000,000 (A currency exchange rate of GBP £1=US $1.28 is applicable) being spent on inhalers in the United Kingdom in just 6 months in 2019 [21] and significant environmental impact. Inhalers alone are responsible for 3% of the National Health Service carbon emissions [22], particularly metered-dose inhalers that use potent greenhouse gases as propellant agents.

There is a need to understand the experiences of patients with BPD and ILO, raise awareness of these conditions, educate clinicians, and provide patient-centered care. Online communities can include people who do not take part in traditional research studies, thereby offering perspectives from an unrepresented patient population [23]. Such data can provide new and insightful perspectives on ILO and BPD symptoms in patients with asthma, with the potential to inform health care interventions [24,25].

The aims of this study were (1) to explore whether symptoms of ILO and BPD were topics of discussion in a UK asthma online community; (2) to describe experiences of symptoms consistent with BPD and ILO in participants with asthma; (3) to explore experiences of diagnosis and treatment of these symptoms, in particular, their use of reliever inhalers; and (4) to explore patient experiences of clinician attitudes to BPD and ILO symptoms.

## Methods

### Overview

A qualitative thematic analysis [26] of existing posts on Asthma UK online [27] with over 18,000 users was carried out as described in other studies based on the same data source [28-30].

### Ethics Approval

Ethical approval was sought and granted by the Queen Mary University of London Research Ethics Committee (QMERC22.015). Permission for the use of the community data in this study was obtained from the Asthma+Lung UK charity, and its host platform, HealthUnlocked. Online community users could choose to either restrict posts or questions to users of the particular HealthUnlocked community, send private messages, or share it with others on the web. Only posts that were shared publicly on the web were collected for this study.

To protect the privacy of the community users, only posts made publicly available by the authors were retrieved and analyzed as part of this study. To further protect the intellectual property of the participants, no posts are directly quoted. Instead, particularly insightful posts have been paraphrased when referenced in the study. This approach was chosen over describing the quotes in the third person to avoid depersonalizing the participants’ experiences.

### Data Collection

Google search engine was used to look for relevant posts within the Asthma UK community platform [31]. A scoping exercise was conducted, and different keywords were tested. Such attempts revealed that relevant posts were retrieved when the key terms “blue inhaler” and “breath” were searched together. The threads of discussions for each selected post were analyzed in detail. Posts located chronologically before or after the selected posts were added to the analysis. Username, names, or pseudonyms; sex; age; whether a participant was a person with asthma or a patient discussed by a third party; and third-party relation with the patient (eg, parent) were retrieved where available within the posts. The posts retrieved in this search were analyzed further to look for mention of symptoms of BPD or ILO. For this purpose, a list of key symptoms was developed (see Multimedia Appendix 1), based on the background literature reviewed in advance of the study. This included cough or breathlessness without wheeze, throat tightness, and abnormal breathing patterns noticed by participants including yawning, deep sighing, and hyperventilating, among others. Only posts that mentioned 1 or more of these key symptoms were included in the study. A date range of January 2018 to February 2022 was chosen to limit the collection and analysis of relevant data identified. Although the COVID-19 pandemic and illness were expected to impact the results, these were not discussed within the threads identified by our search. No attempt was made to distinguish between ILO and BPD symptoms, as there is a significant overlap between the symptoms.

### Data Analysis

Relevant posts and their threads were copied and pasted into an Excel (Microsoft Corp) spreadsheet, and usernames were replaced with unique study IDs for qualitative thematic analysis [26]. Codes related to symptoms, diagnosis, and treatment were created using NVivo software (QSR International). A random sample representing 10% of the data was double-coded by a second researcher (ADS). The coding was discussed and agreed.

Study participants were defined as all users whose entries were included in the study. Demographics including age, gender, and roles within the community were recorded when shared by participants.

## Results

### Overview

**Figure 1:**
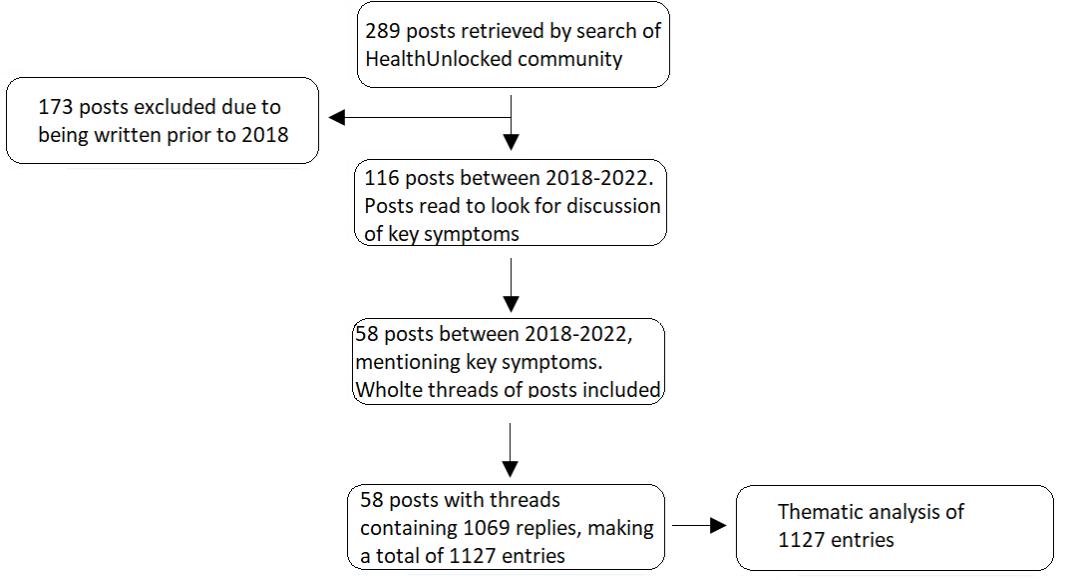
Flowchart summarizing the results of the study search.

The study search resulted in 289 posts, with 116 falling between 2018 and 2022. Fifty-eight (50%) of these were judged relevant for the research questions and included in the analysis. The threads of the 58 included posts contained a total of 1069 other posts, which were also included, making a total of 1127 entries in the data set. These findings are summarized in a flowchart (Figure 1).

### Participants

In total, 1069 of 1127 entries were written by 302 participants, who were identified through their usernames. The remaining 58 entries were written anonymously. All participants were adults writing about their experiences, except 2 who were parents writing about their children. Gender was stated in 5 cases (1.66%; 3 females and 2 males). Age or an age range was declared by 30 participants (9.93%), who were between 27 and 73 years old, with the exception of a 4-year-old child discussed by their parent. The number of entries per participant ranged between 1 and 63, with the average number/participant being 3.54. Seven participants were considered to be very active users (superusers), each of them contributing with >20 posts.

### Themes

#### Overview

A wide range of themes were generated during the analysis. These are reported in Textbox 1.

The findings for each theme are discussed below, supported by relevant quotes from the data. Age ranges and genders have been specified where data were available.

**Experiences of patients with symptoms that could indicate breathing pattern disorder or inducible laryngeal obstruction: themes and subthemes.**

**Symptoms not typical of asthma**

- Abnormal breathing pattern
- Difficulty inhaling
- Cough without wheeze
- Shortness of breath without wheeze
- Normal peak flow or lung function tests
- Atypical triggers
- Chest or throat tightness without wheeze

**Experiences with diagnostic pathways**

- Peers questioning asthma diagnosis
- Health care professionals doubting asthma behind symptoms
- Change in diagnosis
- Request for interpretation of symptoms
- Wanting to prove one had asthma
- Patient questioning diagnosis

**Negative experiences with asthma treatment**

- Recognized overuse of salbutamol (blue) inhalers
- Salbutamol (blue) inhalers not working as expected
- Asthma treatment other than salbutamol not working as expected
- Uncertainty about using salbutamol (blue) inhalers
- Discussions around overuse of salbutamol (blue) inhalers
- “Toughing it out”

**Emotional burden**

- Embarrassment
- Fear
- Desperation
- Feeling like a fraud
- Guilt

**Negative experiences with health care professionals**

- “One-size-fits-all” approach with asthma
- Distrust in health care professionals
- Feeling unsupported by health care professionals
- Judgment from health care professionals
- Health care professionals not listening

**Positive online peer interactions**

- Seeking shared experiences
- Gratitude for peer support
- Peer empathy
- Signposting to Asthma+Lung UK or British Lung Foundation
- Signposting to the general practitioner or practice asthma nurse
- Following peer advice

**Diagnosis with breathing pattern disorder and inducible laryngeal obstruction**

- Breathing exercises helping
- Postnasal drip
- Reflux
- Online peers suggesting breathing pattern disorder or inducible laryngeal obstruction
- Health care professionals suggesting breathing pattern disorder or inducible laryngeal obstruction
- Online peers advising breathing exercises

#### Symptoms Not Typical of Asthma

Cough and shortness of breath without wheeze were commonly mentioned, as was throat tightness, and many described experiencing several of these symptoms. Participants also discussed symptoms that were not accompanied by a drop in peak flow or inconsistent with asthma such as normal spirometry and lung function tests.

I don’t normally wheeze, but I do cough and my main problem is breathlessness … I had lung function tests that showed I was above average for my sex and height!

Participant #69, aged 35-40 years

Some participants reported symptoms associated with an unusual breathing pattern, such as hyperventilating, gasping, or excessive yawning, which can be common in BPD. Participants discussed “unusual” triggers of their symptoms, which were atypical for asthma but could be indicators of ILO, such as odors [17].

I have a real problem with spice aromas. I was out in my garden earlier, and caught a whiff of something that smelled like curry, and half an hour later I was coughing, short of breath and had a tight chest so I needed my inhaler.

Participant #183

Some participants had noticed new, different symptoms, in addition to longstanding typical asthma symptoms. Acknowledging 2 distinct sets of symptoms led participants to question their diagnosis.

#### Experiences With Diagnostic Pathways

A common reason for engaging with the community was requesting an interpretation of symptoms that users did not feel fitted with the asthma diagnosis they had been given.

*I was diagnosed with asthma years ago, although I was never formally tested and I’ve never really been treated for asthma. I’ve never really believed that it’s asthma though. …I guess I’m asking whether you think this is asthma, and what treatment you think might help me? Thanks.* [Participant #84]

Users also questioned peers’ asthma diagnoses, highlighting which symptoms were atypical for asthma in their reasoning.

Lots of things mimic asthma symptoms, like cough, chest tightness, breathlessness etc, but asthma treatment doesn’t help them because they aren’t really asthma. Given that your recent steroids didn’t help and your inhaler doesn’t really help either, maybe you should look into other causes.

Participant #17

I use my regular medication, but still need my blue inhaler every day. My peak flow doesn’t really drop though. Today I used my blue inhaler more than 30 times, and I’ve used it at least 10 times every day for the last week. I have a GP appointment next week, but just focusing on getting through the weekend.

Anonymous Participant

Some participants described that although they were convinced they had asthma, their health care professionals disagreed. Others had been given an asthma diagnosis and then formally had their diagnosis changed years later.

The nurse said I don’t wheeze enough for it to be asthma … I’ve considered it might be silent reflux, but I’m really hoping it’s just uncontrolled asthma. Hopefully one day I’ll find a doctor who will give me the right treatment.

Participant #92

#### Negative Experiences With Asthma Treatment

Symptoms not relieved by blue (short-acting beta-agonist) inhalers were discussed at length.

Using my reliever makes me more breathless. When I was taking it frequently, I got worse and worse, until they told me my peak flow was good and taking so much inhaler was what was making me breathless, so I actually needed less.

Participant #159

Some participants expressed that other asthma treatments had not helped them, including nebulized salbutamol, preventer inhalers, and oral steroids.

I ended up in A&E with breathing problems and palpitations and was given a blue inhaler. It didn’t do anything at all, so my GP gave me a brown one as well, which didn’t help either. Now I’m on a pink inhaler – that helped for about 3 days, and I felt like I could breathe again, but then it stopped working.

Participant #67, aged 35-40 years

Participants struggled to identify when to use their inhalers, and some were clearly overusing them, describing the negative side effects of salbutamol overuse.

I still struggle with using my blue inhaler … How do know when you need to use it? Right now, my chest feels tight and I kind of feel like I’m breathing through a straw, BUT I have no wheeze or breathlessness, so am I supposed to use the blue?

Participant #1

Although reluctance to seek help was a theme, many users had experienced emergency department visits, hospital admissions, and courses of adjunctive treatment as well as high salbutamol usage.

My preventer doesn’t work for me, so I still have to use 10 puffs a day of my blue inhaler. Even then, I get a severe episode every month or so when I have to go to hospital and have nebulisers and then a course of steroids. It helps, but then I have another attack.

Participant #299, aged 40-45 years

#### Emotional Burden

Participants described negative emotions and frustration as a result of their breathing symptoms not typical of asthma or not responding to asthma treatment.

When things get bad people tell me to relax, but I can’t possibly relax when I feel like I’m about to die!

Participant #114

Countless times I’ve woken up unable to breathe, and it’s terrifying! I try not to panic, and I keep my blue inhaler by the bed so I try to reach for it calmly, but actually I end up desperately grabbing for it in panic, because I’m scared!

Participant #81

They struggled to convince others of the reality of their pain.

I try explaining to my friends and family that when every single waking breath you take is a struggle and you can’t get a good breath in, it’s incredibly frightening, debilitating, distracting and enraging.

Participant #48

Participants also felt fear, particularly over the sensation of not being able to inhale, and desperation, “feeling like a fraud” for having unexplained symptoms.

I’m sure if I ask my asthma nurse they’ll say I’m fine because my peak flow isn’t low … even when I struggle my peak flow is within the green zone … I wish it would reflect my symptoms, it’s infuriating. Is it all in my head? I feel like a fraud.

Participant #128

I feel like I’m a fraud, or making things up etc. Plus I know there are loads of people with asthma and other lung diseases who have it way worse, so I feel like I should just be able to get on with it, and I get exasperated with myself for getting worked up like this.

Anonymous Participant

#### Negative Experiences With Health Care Professionals

On the whole, participants displayed negative perceptions of health care professionals (HCPs), reporting feeling judged for their unusual breathing symptoms.

I’ll warn you, I was diagnosed [with BPD] by a respiratory specialist, but a lot of health professionals still laugh and ask me how I can forget to breathe.

Participant #105

It’s really difficult not knowing where you stand and being told you’re a hypochondriac, that it’s just the way you breathe, or you aren’t trying hard enough, and you don’t know what’s happening or how to make it better, especially if you’re usually healthy and active.

Participant #22

Some felt that HCPs were not listening to them or were not taking them seriously.

My asthma nurse asked me if I was sure my cough wasn’t just a habit!?

Anonymous Participant

Suggestions were made that doctors use a “one size fits all” approach to managing asthma, overly focusing on wheezing as an indicator of asthma.

I don’t think the hospital would take me seriously, because all my test results are normal … my GP and asthma nurse say there’s no need to repeat the tests … I feel like I’m trying to prove that there’s something wrong.

Participant #33

Participants had been told by HCPs that nothing could be done about symptoms that troubled them.

I dread going to see my GP because they don’t understand what I mean when I say I’m breathless, and I’m not very good at standing up for myself or explaining it … the doctors think it’s because I’m not compliant with my preventer, but I am … The respiratory consultant says my cough is caused by postnasal drip and made worse by me thinking it’s a chronic thing, and that my breathlessness and tight chest are “a perceived feeling” with no actual cause.

Anonymous Participant

#### Positive Online Peer Interactions

Participants repeatedly sought others in similar situations and discussed their shared experiences, which they stated brought them comfort and relief.

I wouldn’t wish this on anyone, but it’s still really comforting to know there are others with the same strange symptoms that don’t seem to “meet the criteria” for a diagnosis.

Participant #126

Participants also offered empathy.

My situation is so much like yours! Sorry I’m not able to give advice, but it’s so good to know I’m not the only one dealing with this!

Participant #100

I know exactly how you feel – you aren’t alone. I’m living with symptoms exactly the same as yours. Although it hardly feels like living.

Participant #196

Others posted well wishes to online peers,

Everything you’ve said is valid, and we’re all happy to listen any time. You must feel so frustrated not knowing what the problem is, and I know all of us sometimes need to vent too. Other people have been in your shoes, regarding not having a diagnosis. Look after yourself, and keep us updated.

Participant #135, aged 60-65 years

A few participants offered advice including signposting to the GP or to resources such as Asthma UK.

If you want, you can call the Asthma UK nurses to talk about your symptoms and treatment. Here are their contact details. They can help you plan what you’re going to ask your doctor for.

Participant #65

Thank you. I’m so grateful for this forum. I don’t know what I’d do without it.

Participant #1

Participants were grateful for the peer support and returned to the community to share that they had followed, or planned to follow, the advice peers had given them. As a result of engagement with the online community, some participants went back to their clinicians and were eventually able to get an ILO diagnosis.

I just wanted to give you all an update. I followed your advice and saw my doctor, who referred me to a lung specialist. She was great and actually listened to me, and has diagnosed me with intermittent laryngeal obstruction! It means my larynx narrows and makes it hard for me to breathe, but it doesn’t cause wheezing.

Participant #283

#### Diagnosis With BPD and ILO

Some participants had been formally diagnosed with BPD or ILO, with most reporting feeling positive about these diagnoses.

Your situation sounds very similar to mine, and I’ve been diagnosed with dysfunctional breathing pattern. It’s essentially where your body breathes quickly and shallowly without realising it, and sometimes you can hold your breath subconsciously too. It sounds insane, but once it becomes normal for you your body isn’t able to do things that require good breathing, like exercise. Physiotherapy can really help though. They can help you relearn to breathe normally, and give you exercises to practice as well.

Participant #105

They were keen to explain to other users what exactly the condition was and how the symptoms can mimic asthma.

I’ve been having difficulties with shortness of breath. I went to the ED once, and had a whole load of normal tests. They couldn’t find any reason why I was so breathless. I mentioned that I’ve been referred for an endoscopy to check for vocal cord dysfunction, and they concluded it was probably that causing my breathlessness.

Participant #245

If it feels like the tightness is more in your throat than your chest it could be a good idea to look into VCD/ILO, or a breathing pattern disorder like [Participant 105] suggested. These can both look like asthma, but they can be helped by breathing exercises.

Participant #23

There were also specific suggestions of BPD and ILO by community users, in response to posts where participants discussed symptoms they thought would fit with one of these diagnoses.

Have you heard of inducible laryngeal obstruction/vocal cord dysfunction? Lots of your symptoms sound like they could be caused by that; the fact you’re triggered by laughing and talking, your issues with swallowing and choking/coughing, the shortness of breath and tight chest when breathing in etc.

Participant #23

If your inhalers aren’t helping, I’d ask your doctor to refer you to be investigated for vocal cord dysfunction. It has similar symptoms to asthma, but the treatment is different. I’ve heard it’s a diagnosis that gets missed a lot.

Participant #258

Various types of breathing exercises were recommended for symptom relief, and many participants discussed their own personal success with breathing exercises.

Check out the YouTube video on the Buteyko method. A respiratory physiotherapist recommended it to me, to reduce my cough. Try the technique before the cough gets going.

Participant #217

I was sceptical, but my physiotherapist persuaded me to try breathing exercises and they’ve really helped, although I find them hard work as I have a lot of bad habits … I wish I’d started them years ago.

Participant #65

## Discussion

### Summary of Main Findings

The symptoms typical of BPD and ILO were experienced and discussed by participants in an Asthma UK community. Indeed, some participants were experiencing both asthma and BPD and ILO concurrently. Evidence of salbutamol inappropriate use was also present, particularly in participants whose symptoms did not fit with those listed on a standard asthma action plan, for example, those who described feeling very breathless but had normal peak flows. The results show awareness of salbutamol overuse in undiagnosed BPD and ILO among some participants.

Misdiagnosis and unclear diagnosis were sources of frustration, and experiencing persistent debilitating symptoms that do not seem to fit with an asthma diagnosis left some participants feeling like “frauds.”

The suggestions from HCPs regarding abnormal breathing pattern being the cause of symptoms could be met by participants’ frustration, and participants could perceive such suggestions as criticism. On the other hand, participants formally diagnosed with BPD or ILO were positive and seemed to welcome their diagnoses. Participants were keen to help others by picking out symptoms inconsistent with asthma and giving their opinions on contacting back their clinicians for possible alternative diagnoses in a supportive and nonconfrontational manner.

Interestingly, there seemed to be knowledge among some participants about BPD and ILO, and more generally of different causes of coughing than asthma, such as gastroesophageal reflux disease [12] and postnasal drip, both of which are potential triggers of ILO [15].

Perceptions of HCPs could be negative, particularly when participants felt unsupported and not listened to, partly due to lack of awareness of BPD and ILO, meaning low rates of diagnosis, and therefore inappropriate management.

There was evidence that participants found breathing exercises effective and that online resources could be an effective way to educate patients on the techniques and how or when to use them. Links to online resources in general seemed well-liked, well-used, and are clearly a valuable resource.

Engaging with the community allowed participants to receive emotional and behavioral support [32,33], triggering action in some, who reported contacting back their clinicians and reaching a diagnosis of ILO. This is an example of a reported patient behavior change through engagement with an online health community [34].

### Interpretation of Findings in the Context of Existing Evidence

Results presented here confirm and expand previous evidence that BPD and ILO symptoms are often attributed and misdiagnosed as asthma [3,4,12,13], resulting in poor control of symptoms [13]. Inappropriate use of salbutamol and its negative consequences such as psychological dependence on inhalers [18,19] and side effects were also reported. The emotional burden of patients with BPD and ILO symptoms was an important theme. The day-to-day life of many of these patients is significantly impacted by their symptoms, in keeping with the evidence that patients with BPD and ILO have more mental health illnesses and lower quality of life than those with asthma alone [35,36].

The community itself was a valuable resource for patients for seeking support and advice. Engaging with peers with shared experiences was particularly important to participants, reflecting research findings which suggest that sharing personal experiences is a key benefit of using online communities [30,37-40].

### Strengths and Limitations

A main strength of this work was the use of an established online community. Results were based on participants’ agendas and allowed views to be identified that may not have been captured otherwise, from a wide geographical location.

This study has several limitations. While every one of the posts identified through the keyword search mentioned key symptoms that led to inclusion in the study, not all replies mentioned them. There were replies where members elaborated on their situations without rementioning key BPD and ILO symptoms. Other replies offered support to others or simply reflected on the situation being discussed. Some participants described their own symptoms, which could be in-keeping with classical asthma and some engaged in replies without ever describing whether they were experiencing similar key symptoms. However, all entries were included to preserve the richness of the data of whole conversations. Therefore, neither the number of entries nor the number of participants in this study directly correlates to the number of patients with key symptoms, which could not be calculated.

The key terms “breath” and “blue inhaler” worked well for finding relevant posts, though may have missed instances where other terms were used. Nonetheless, a saturation of themes was reached with the posts identified.

Little data were available within the posts on demographics such as age, gender, and asthma severity.

The method of participant selection for this study potentially resulted in a participant pool that may be less diverse than the general population. Patients without access to a computer, those unable to read and write, those who did not speak English, and those who lacked the language or social skills to be able to engage meaningfully with an online community were not able to be included.

Finally, the authenticity of the forum content could not be assessed. Moreover, the online health community was moderated and some of the posts might have been removed or affected by the moderation process.

### Conclusions

This study shows that patients experiencing BPD and ILO, conditions with an estimated prevalence of one-third to one-fifth of all patients with asthma, have unmet needs. Participants expressed frustration with the “one-size-fits-all” approach to diagnosis and were aware of their salbutamol overuse due to the lack of effect on symptoms. The asthma online community was a valuable resource, and there was qualitative evidence of engagement prompting patients’ action and behavior change. Further education and training for clinicians on BPD and ILO diagnosis and management, particularly in general practice, is needed. Widening the availability of appropriate management options, such as respiratory physiotherapy and speech and language therapy, could also improve care for patients with BPD and ILO.

Considering their clear value to patients, there may be benefits in further development of BPD and ILO online resources and encouraging engagement in an online asthma community.

## Appendix

Multimedia Appendix 1 Key symptoms used to determine the relevance of the post.

## Abbreviations

BPD: breathing pattern disorder
HCP: health care professional
ILO: inducible laryngeal obstruction

## Data Availability

This is not applicable, as data were not collected for this study and are currently available on the internet. Asthma UK and HealthUnlocked have approved the study protocol.
