# Supplementary material for: Experiences of Diagnosis, Symptoms, and Use of Reliever Inhalers in Patients With Asthma and Concurrent Inducible Laryngeal Obstruction or Breathing Pattern Disorder: Qualitative Analysis of a UK Asthma Online Community
Source: J Med Internet Res. 2023 Aug 14;25:e44453. doi: 10.2196/44453 (PMC10463086; doi:10.2196/44453)
Supplement: Multimedia Appendix 1 [file jmir_v25i1e44453_app1.docx]

**Table S1.** The key symptoms used to determine relevance of post.

| Key Symptoms of BPD and ILO |
| --- |
| Abnormal breathing rhythm that the patient is aware of |
| Chest or throat tightness without wheeze |
| Cough without wheeze |
| Difficulty Inhaling |
| Dysphonia or hoarse voice |
| Shortness of breath without wheeze |
| Symptoms triggered by odors or vapors |
